# Modeling environmental risk factors of autism in mice induces IBD-related gut microbial dysbiosis and hyperserotonemia

**DOI:** 10.1186/s13041-017-0292-0

**Published:** 2017-04-20

**Authors:** Joon Seo Lim, Mi Young Lim, Yongbin Choi, GwangPyo Ko

**Affiliations:** 1 Department of Environmental Health Sciences, Graduate School of Public Health, Seoul National University, Seoul, Republic of Korea; 2 Institute of Health and Environment, Seoul National University, Seoul, Republic of Korea; 3 Research Group of Gut Microbiome, Korea Food Research Institute, Seongnam, Gyeonggi-do Republic of Korea; 4 Center for Human and Environmental Microbiome, Seoul National University, Seoul, Republic of Korea; 5 N-Bio, Seoul National University, Seoul, Republic of Korea; 6 KoBioLabs, Inc., 1-Gwanak-ro, Gwanak-gu, Bldg 220, Rm 630, Seoul, 151-746 Republic of Korea

## Abstract

Autism spectrum disorder (ASD) is a range of neurodevelopmental conditions that are sharply increasing in prevalence worldwide. Intriguingly, ASD is often accompanied by an array of systemic aberrations including (1) increased serotonin, (2) various modes of gastrointestinal disorders, and (3) inflammatory bowel disease (IBD), albeit the underlying cause for such comorbidities remains uncertain. Also, accumulating number of studies report that the gut microbial composition is significantly altered in children with ASD or patients with IBD. Surprisingly, when we analyzed the gut microbiota of poly I:C and VPA-induced mouse models of ASD, we found a distinct pattern of microbial dysbiosis that highly recapitulated those reported in clinical cases of ASD and IBD. Moreover, we report that such microbial dysbiosis led to notable perturbations in microbial metabolic pathways that are known to negatively affect the host, especially with regards to the pathogenesis of ASD and IBD. Lastly, we found that serum level of serotonin is significantly increased in both poly I:C and VPA mice, and that it correlates with increases of a bacterial genus and a metabolic pathway that are implicated in stimulation of host serotonin production. Our results using animal model identify prenatal environmental risk factors of autism as possible causative agents of IBD-related gut microbial dysbiosis in ASD, and suggest a multifaceted role of gut microbiota in the systemic pathogenesis of ASD and hyperserotonemia.

**Electronic supplementary material:**

The online version of this article (doi:10.1186/s13041-017-0292-0) contains supplementary material, which is available to authorized users.

## Introduction

Autism spectrum disorder (ASD) is characterized by core deficits in neurodevelopmental milestones. However, a majority of children with ASD also suffer from a wide range of systemic aberrations such as increased serum serotonin (40% prevalence) [1–3], gastrointestinal (GI) distress (up to 90%) [4], and inflammatory bowel disease (IBD) [5]. Unfortunately, the causative agent for those conditions and their relationship with etiology and pathogenesis of ASD remains undefined.

Gut microbiota carries a central role in the health status of its host via regulation of immune system and metabolism [6]. Intuitively, one of the most directly affected host site by microbial dysbiosis is the GI tract, in that dysbiosis is responsible for the development of GI disorders and IBD [7]. Gut microbiota is also able to affect the host’s neurodevelopmental status via gut-brain-axis [8]. Importantly, a recent paper demonstrated that administration of beneficial bacteria can ameliorate a subset of behavioral abnormalities in a mouse model of autism [9]. Moreover, gut microbiota profoundly impacts host serotonin production [10], an imbalance of which leads to GI discomfort and altered mental status [11].

Co-occurrence of two or more mutually exclusive, etiologically unrelated diseases is rare; therefore, the fact that GI disorders and IBD are so prevalent among children with ASD is highly suggestive of a common cause for the behavioral abnormalities and systemic aberrations in ASD. Accordingly, accumulating number of studies report that the gut microbial composition and several microbial metabolic pathways are significantly altered in children with ASD [12, 13] as well as patients with IBD [14, 15].

The rising global incidence of ASD [16] implies that environmental factors might be a contributing component in its etiology. We therefore hypothesized that environmental risk factors are responsible for the gut microbial dysbiosis in ASD, and that such dysbiosis is a driving force for the wide range of systemic aberrations in ASD. To test this, we chose prenatal injection of polyinosinic:polycytidylic acid (poly I:C) and valproic acid (VPA) as our model, which represent two of the most widely utilized environmental risk factors of ASD. We analyzed the overall composition of gut microbiota in poly I:C and VPA mice as well as the relative abundances of 259 microbial taxa and compared the results to existing reports on microbial dysbiosis in clinical cases of ASD and IBD. We also performed network analysis to examine the correlation between microbial families, and applied the relative abundance data to metabolic pathway database in order to assess how microbial dysbiosis might affect the host’s metabolic system in ASD. Lastly, we tested if ASD mice also display hyperserotonemia, another highly occurring comorbidities in ASD.

## Methods

### Generation of environmental risk factor-induced mouse models of autism

Pregnant C57BL/6 mice from Japan SLC, Inc. (Japan) were purchased via Central Lab. Animal Inc. (Seoul, Korea) and housed in animal biosafety level 2 SPF facility at Seoul National University College of Medicine, Korea, with ad libitum supply of standard chow and water. At embryonic day 12.5 (E12.5), a total of 15 pregnant dams were intraperitoneally injected with poly I:C (20 mg/kg; Sigma-Aldrich, USA), valproic acid (VPA) (500 mg/kg; Sigma-Aldrich, USA), or equivalent amount of saline (5 dams per group). The pups were weaned at three weeks after birth and were supplied with the same kind of chow and water. Only male pups were used for all subsequent experiments.

### Behavioral assay

Poly I:C and VPA offspring were subjected to behavioral assays relevant to autism phenotype at six weeks of age. The mice were first tested for anxiety-related behavior using open field chamber (42*42*42 cm), in which exploratory behavior in a novel environment was assessed in 10-minute sessions. Locomotor activity was captured with a video camera and analyzed with Ethovision XT software (Noldus, USA). The amount of total movement during the experimental session as well as time spent in the center region (30*30 cm area) was measured and compared to corresponding control (CTL) group.

The mice were also tested for social interaction behavior using 3-chamber assay [9]. Briefly, testing mice were habituated for 10 min in the chamber; then two cylindrical cages were placed on each side of the chamber, one of which contained a stranger (mouse of the same sex and age raised in a separate cage), thus testing for social preference over the non-social cue. 10 min after the initial interaction, a second stranger mouse was placed in the empty cage and the behavior of the testing mouse was again recorded for 10 min, which represents a preference for a novel social cue. The relative time spent on each cylindrical cage during each experimental phase was calculated and compared to that of CTL mice.

### Serum serotonin measurement

Blood samples were collected from submandibular veins of mother and offspring mice by using Goldenrod Animal Lancet (Braintree Scientific, Inc., USA) according to the manufacturer’s instructions. Serum was separated from whole blood by allowing the blood to clot at room temperature, then centrifuging at 1,000 x g for 10 minutes at 4 °C. The resulting serum supernatant was then stored in −20 °C until use. Serum levels of serotonin were measured using commercial ELISA assay (Eagle Biosciences, USA) according to the manufacturer’s instructions, and values from poly I:C and VPA groups were compared to the corresponding CTL groups of the same age in both mother and offspring mice.

### DNA extraction and Illumina sequencing of mouse fecal DNA

Fecal samples from mother and offspring mice were collected at indicated time points and immediately placed in liquid nitrogen. DNA was extracted from feces using QIAamp DNA Stool Mini Kit according to the manufacturer’s instruction (Qiagen, USA), and stored in −20 °C until use. The fecal DNA samples were PCR-amplified using Illumina-adapted universal primers 515 F and 806R that target the V4 region of the 16S rRNA gene [17], and the amplicons were quantified with the KAPA Library Quantification Kit (KAPA Biosystems, USA). The resulting amplicons from each sample were sequenced using the MiSeq platform (Illumina, USA), which yielded 16S rRNA data for further analysis.

### 16S rRNA gene sequence processing and statistical analysis

16S data from mice fecal DNA were processed and analyzed using QIIME 1.8 software package [18]. First, the sequences were clustered into operational taxonomic units (OTUs) at 97% identity using OTU-picking protocol, and the relative abundances of microbial taxa from genus to kingdom were generated from non-rarefied OTU table. Alpha-diversity indexes (PD index, Shannon diversity index) were estimated and tested for significant differences between CTL and poly I:C or VPA groups using Monte Carlo permutations. Beta-diversity using unweighted UniFrac distance was calculated onto a rarefied OTU table, which was grouped into either timing of sampling or drug treatment.

To evaluate the co-occurrence relationships between microbial taxa, we calculated Spearman’s ranked correlation of their relative abundances, and the networks of co-occurring taxa were then visualized using Cytoscape [19, 20]. Each node represents a microbial family (e.g., Prevotellaceae) and the nodes were paired when their q-values and p-values were both below 0.05, respectively. The length of the linkage is proportional to the closeness of the two families. Microbial functions from the 16S data were predicted by using PICRUSt [21] and collapsing the predicted functions into higher categories according to Kyoto Encyclopedia of Genes and Genomes (KEGG) orthology as previously described [22], and the resulting functioning profiles were visualized as a heatmap using R package [23].

Statistical significance of differences between CTL and poly I:C or VPA groups in each experiment were calculated using either R or Microsoft Excel 2013 using paired t-test. Differences between groups were determined significant at *p < 0.05, **p < 0.01, ***p < 0.001, and ****p < 0.0001.

## Results

### ASD mice have reduced gut bacterial diversity

According to previous literature on generating environmental risk factor-induced ASD model, we intraperitoneally injected pregnant C57BL/6 mice with 20 mg/kg poly I:C [9, 24] or 500 mg/kg VPA [25] at embryonic day 12.5 (E12.5). When subjected to a battery of standard behavioral testing at six weeks of age, both poly I:C and VPA offspring, hereafter referred to as ASD mice, showed autism-related behavioral abnormalities including increased anxiety and decreased social behavior (Additional file 1: Figure S1).

**Fig. 1:**
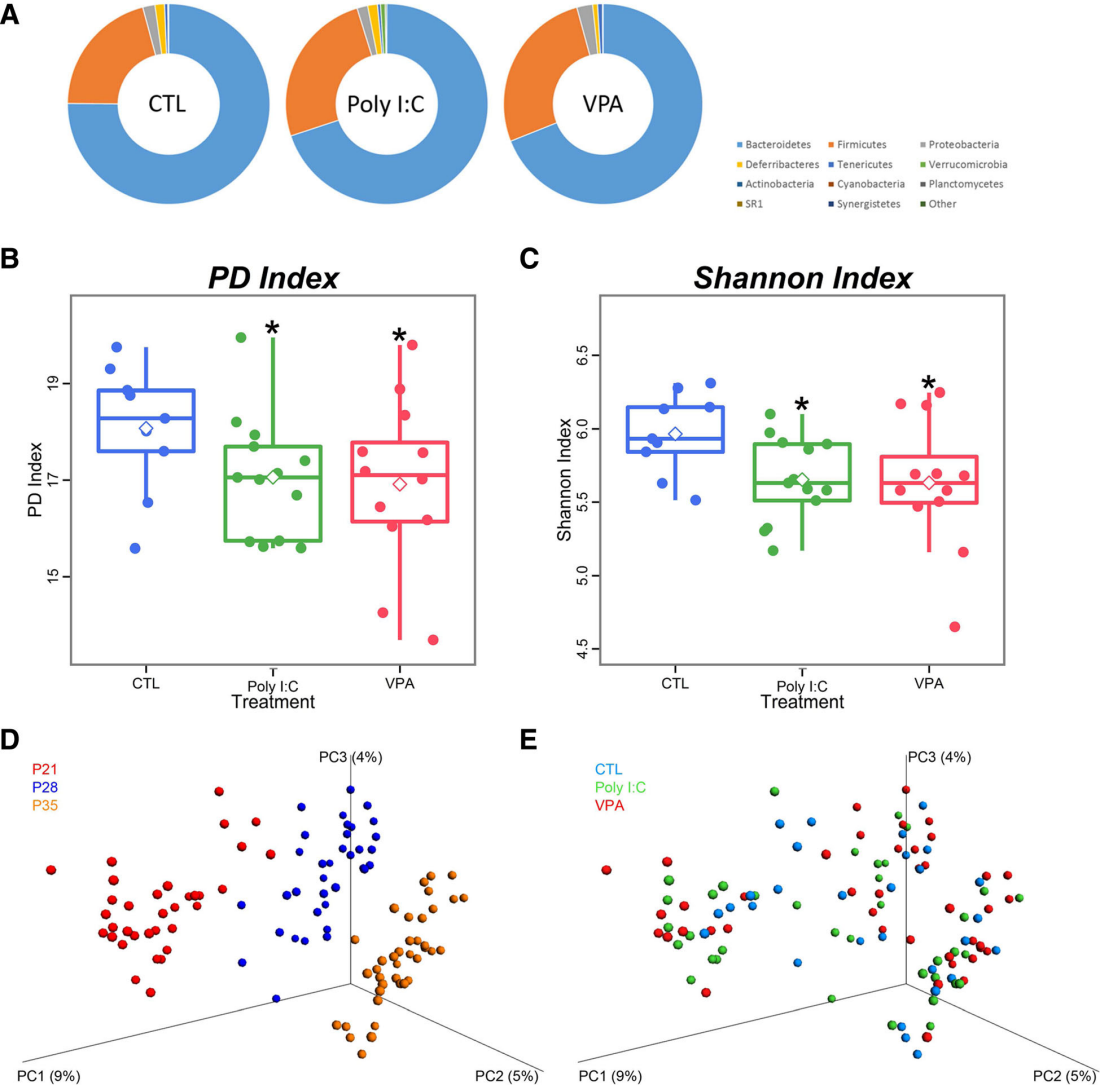
Gut microbial composition and diversity indexes in ASD mice. **a** Gut microbial composition comparison at the phylum level. **b** PD index and **c** Shannon index of alpha-diversity in CTL and ASD mice. *p < 0.05 vs. CTL. Beta-analysis of CTL and ASD mice shown by **d** timing of feces sampling or **e** drug treatment. *n* = 9–14 in all experiments

We analyzed the composition of fecal microbiota of post-weaned postnatal day 21 (P21) mice, and observed that the overall composition of gut microbes of ASD mice did not significantly differ from that of CTL mice at the phylum level, except for a slight increase in Firmicutes accompanied by a roughly equal amount of decrease in Bacteroidetes (Fig. 1a). On the other hand, analysis of two alpha-diversity indexes (PD index and Shannon index) showed that ASD mice harbor significantly less diverse microbial species (Figs. 1b and c), which is in line with previous findings in children with ASD [13].

### Dietary change, but not poly I:C or VPA, induces beta-diversity shifts in mice

Beta-diversity index measures the compositional difference of microbial community between experimental groups [26], and denotes if a general shift in the microbial community occurred in response to a stimulus, which in this case was ASD induction. We analyzed fecal samples from CTL and ASD mice right before weaning at P21, and twice more in one-week intervals (P28, P35). Unweighted beta-diversity analysis showed that as the mice went through dietary change from mother’s milk (P21) to standard chow (P28, P35), their gut microbiota experienced a distinguishable shift in their composition (Fig. 1d). However, in each of those time points, the microbial communities of ASD mice were not clearly distinguished from that of CTL mice (Fig. 1e). Collectively, the gut microbiota of ASD mice was not distinguishable from that of CTL mice at the phylum level or in their general composition; rather, these results suggest that the decrease in their alpha-diversity indexes might stem from dysbiosis at the genus or species level.

### ASD- and IBD-related gut bacterial species are altered in ASD mice

**Fig. 2:**
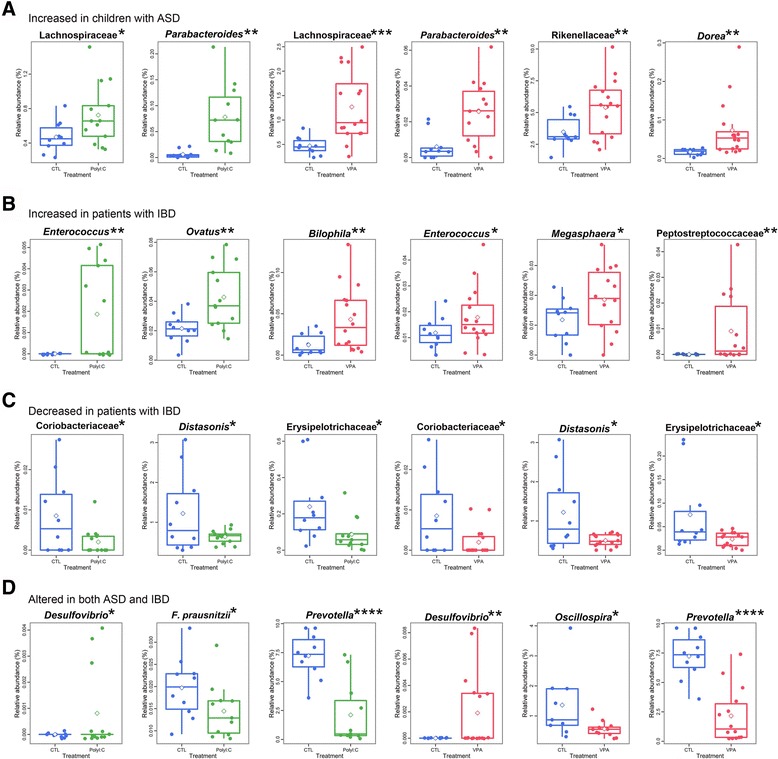
Relative abundances of gut microbial taxa in ASD mice. Relative abundances of microbial taxa in CTL and ASD mice. The data are divided with respect to the pattern of dysbiosis reported in previous clinical studies on either children with ASD or patients with IBD. **a** Taxa reported to have been significantly **a** increased in children with ASD or **b** patients with IBD. **c** Taxa that are decreased in patients with IBD. **d** Taxa that are increased (*Desulfovibrio*) or decreased (*Prevotella*, *F. prausnitzii*, *Oscillospira*) in both ASD and IBD. Asterisks next to taxa names denote the statistical significance of the difference between CTL and each ASD experimental group. *p < 0.05, **p < 0.01, ***p < 0.001, ****p < 0.0001 vs. CTL. n = 9-14 in all experiments

A number of studies have reported that children with ASD [12, 13, 27–29] or patients with IBD [15, 30–39] have altered gut microbial composition at the genus or species level, some of which are consistently reported across a number of clinical studies (summarized in Table 1).We therefore performed relative abundance analysis of gut microbiota of ASD mice at various taxonomic levels ranging from phylum to species, and found that prenatal injection of poly I:C and VPA resulted in significant changes in specific microbial taxa in a pattern that highly recapitulates those of clinical ASD and IBD (Fig. 2). Both poly I:C and VPA mice showed significant increases in bacterial species that are abundant in ASD (Fig. 2a) or IBD (Fig. 2b), including *Enterococcus* [40], which has been suspected to have a causal relationship with the disease progression of IBD. ASD mice also had significantly less abundant species that are decreased in clinical IBD (Fig. 2c), and in the case of *Desulfovibrio*, both the children with ASD and Crohn’s disease patients had increased abundance of the genus, a pattern also observed in ASD mice (Fig. 2d). Lastly, ASD mice had decreased abundance of *Oscillospira sp*., *F. Prausnitzii*, and *Prevotella*—all of which are underrepresented in clinical cases of ASD and IBD (Fig. 2d).

**Table 1:** Bacterial taxa reported as altered in clinical cases of ASD and IBD

	Bacteria	vs. HC	Reference
ASD-related taxa	*Dorea sp.*	↑	[12]
	*Parabacteroides*	↑	[28]
	***Desulfovibrio***	**↑**	[28]
	***Oscillospira sp.***	**↓**	[12]
	***F. Prausnitzii***	**↓**	[12]
	***Prevotella***	**↓**	[12] [13],
IBD-related taxa	*Bilophila*	↑	[30]
	*Enterococcus*	↑	[32]
	*Megasphaera*	↑***	[33]
	*Bacteroides ovatus*	↑	[34]
	Peptostreptococcaceae	↑**	[35]
	Coriobacteriaceae	↓*^,^ **	[50]
	*P. distasonis*	↓	[78]
	Erysipelotrichaceae	↓*	[36]
	***Desulfovibrio***	↑**	[37]
	***Oscillospira sp.***	↓*	[38]
	***F. prausnitzii***	↓*	[79]
	***Prevotella***	**↓**	[15]

Bold taxa are reported to be altered in both ASD and IBD. The asterisks (*, **, and ***) next to the *arrows* denote alterations reported in Crohn’s Disease, ulcerative colitis, and irritable bowel syndrome, respectively

Comprehensive lists of microbial taxa significantly altered in poly I:C and VPA mice are provided in the online version of this manuscript (Additional file 4: Table S1).

### Prevotellaceae family is significantly and independently decreased in ASD mice

**Fig. 3:**
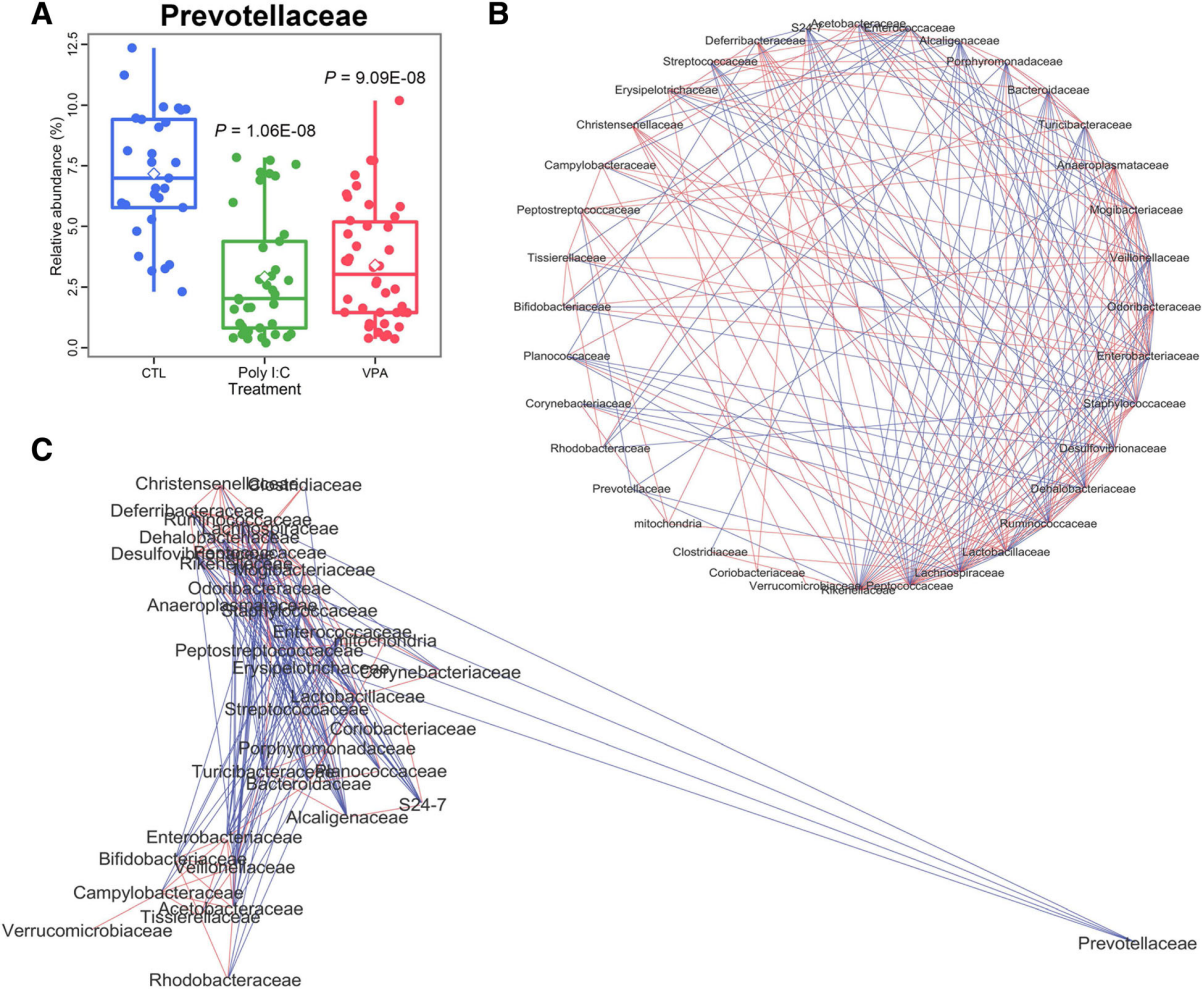
Prevotellaceae family is significantly and independently altered in ASD mice. **a** Relative abundance of Prevotellaceae in ASD mice compared to CTL mice. *P* values were calculated against CTL group. **b** Unweighted co-occurrence network analysis of gut microbial families in ASD mice. **c** Co-occurrence network weighted with linkage strength. *Red* and *blue lines* denote positive and negative correlation, respectively

Out of the 259 microbial taxa tested, the genus *Prevotella* showed the most prominent change compared to CTL, with statistical significance well below *p* < 0.0001 (Fig. 2d) in both poly I:C and VPA mice. When we reanalyzed the gut microbial abundance at the family level, Prevotellaceae comprised more than 7% of the total gut microbial community in CTL mice, whereas it only spanned ~2.5% in ASD mice, with many specimens showing close to zero percentage abundances (Fig. 3a). We then performed co-occurrence analysis based on Spearman’s rank correlations to study how microbial families were interlinked (Fig. 3b), and found that families such as Rikenellaceae, Peptoccaceae, and Lactobacillaceae were significantly correlated to more than dozen other families, indicating that as the abundances of those families change, so do the abundances of other dozen families. However, Prevotellaceae was negatively correlated to only four other families—Rikenellaceae, Lachnospiraceae, Anaeroplasmataceae, and Peptostreptococcaceae; moreover, when we applied weight to the co-occurrence data so that closely linked families will be shown as such, Prevotellaceae was shown to be separated from all the other families by a great margin (Fig. 3c). This result suggests that dysbiosis in other families is not likely to have caused a noticeable shift in the abundance of Prevotellaceae, and vice versa. Taken together, the decrease in *Prevotella*, which has been reported in clinical cases of both ASD and IBD, is highly unlikely to have been brought forth by alterations in other microbial communities in our mouse model, but more likely by an inhospitable host environment due to ASD induction.

### Metabolic pathways implicated in ASD and IBD are altered in KEGG pathway analysis of poly I:C and VPA mice

**Fig. 4:**
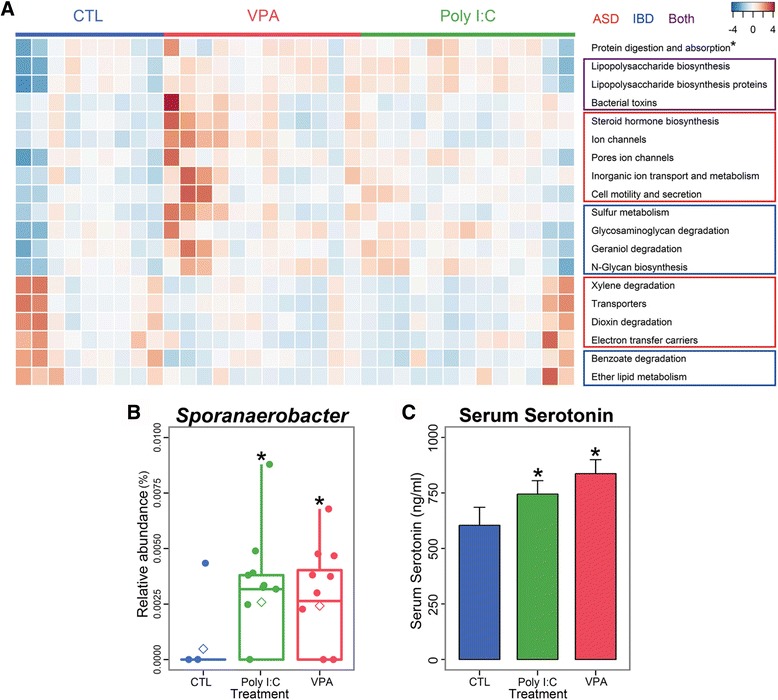
Possible metabolic perturbations caused by microbial dysbiosis in ASD mice. **a** KEGG pathway analysis based on 16S data drawn out in heatmap. All pathways except “Protein digestion and absorption” have significant (*p* < 0.05) differences between CTL and each ASD experimental group. In “Protein digestion and absorption”, the *p*-values of CTL vs. VPA and CTL vs. Poly I:C are 0.045 and 0.055, respectively. Pathways in the *red box* have been reported to be perturbed in children with ASD, and those in the *blue box* have been implicated in patients with IBD. *Purple box* denotes that ASD and IBD showed the same manner of alteration in those pathways. **b** Relative abundance analysis of *Sporanaerobacter* in CTL and ASD mice. *p < 0.05 vs. CTL. **c** Serotonin level in serum detected by ELISA in CTL and ASD mice. *p < 0.05 vs. CTL. *n* = 9-14 in all experiments

We have thus far examined how ASD induction by environmental risk factors leads to clinically-relevant microbial dysbiosis in the offspring. Consequently, we sought to get an insight of how microbial dysbiosis in turn affects the metabolism of ASD mice. We assessed the functional potential of each experimental groups by applying the 16S data into Kyoto Encyclopedia of Genes and Genomes (KEGG) pathway abundances [22]. As a result, we found that many pathways that have previously been implicated in ASD (dioxin degradation [41], steroid hormone biosynthesis [42]) were altered in the same manner in ASD mice (Fig. 4a, Table 2), as well as those implicated in IBD (sulfur metabolism [43], N-Glycan biosynthesis; [44] Table 2). Notably, lipopolysaccharide (LPS) biosynthesis and bacterial toxin have been implicated in the pathogenesis of both ASD [45] and IBD; [46] accordingly, our results showed that both poly I:C and VPA mice have significant up-regulations in pathways involving LPS biosynthesis and bacterial toxins (Fig. 4a).

**Table 2:** Metabolic pathways implicated in clinical cases of ASD and IBD

	Pathway	vs. HC	Reference
ASD-related pathways	LPS/LPS proteins/Bacterial toxins	**↑**	[45]
	Steroid hormone biosynthesis	↑	[42]
	Ion channels/Pores ion channels	↑	[59]
	Inorganic ion transport and metabolism	↑	[60]
	Cell motility and secretion	↑	[61]
	Xylene degradation	↓	[63]
	Transporters	↓	[64]
	Dioxin degradation	↓	[41]
	Electron transfer carriers	↓	[65]
IBD-related pathways	LPS/LPS proteins/Bacterial toxins	**↑**	[46]
	Sulfur metabolism	↑	[43]
	Glycosaminoglycan degradation	↑	[67]
	Geraniol degradation	↑	[68]
	N-Glycan biosynthesis	↑	[44]
	Benzoate degradation	↓	[51]
	Ether lipid metabolism	↓	[69]

Bold pathways are reported to be altered in both ASD and IBD

Comprehensive lists of metabolic pathways significantly altered in poly I:C and VPA mice are provided in the online version of this manuscript (Additional file 5: Table S2).

### ASD mice have elevated level of serum serotonin

Interestingly, we observed one pathway that was up-regulated in VPA (*p* = 0.047) and poly I:C mice (*p* = 0.055), which was not directly implicated in the disease progression of either ASD or IBD: “Protein digestion and absorption (hsa04974)”. Nevertheless, this pathway was of potential interest in that it is implicated in the production of short chain fatty acids (SCFAs), which are known to stimulate serotonin production from enterochromaffin cells [47]. Elevated serum serotonin, along with GI disorders and IBD, is another highly occurring comorbidity in ASD [3]. Importantly, a recent publication has shown that the gut microbes are responsible for stimulating host serotonin production, and noted that spore-forming bacteria of Clostridia class are responsible for such action.

We therefore analyzed the relative abundance of Clostridia class bacteria in ASD mice and found that Clostridiales Tissierellaceae *Sporanaerobacter*, a genus that forms spores and produces SCFAs [48], was significantly increased in both poly I:C and VPA mice (Fig. 4b). The increase in SCFA-producing pathway in KEGG analysis, as well as the increased abundance of spore-forming, acetic-acid producing bacteria *Sporanaerobacter*, indicated that serum serotonin might be increased in these mice as well. Indeed, when we analyzed the serum from ASD mice, we observed a significant increase of serum serotonin level compared to CTL mice (Fig. 4c), a phenomenon not previously reported in poly I:C and VPA mice models of ASD.

Taken together, our results show that microbial dysbiosis induced by prenatal poly I:C and VPA treatment not only recapitulated many aspects of microbial dysbiosis in clinical ASD and IBD, but may also have functional properties that affect the host’s health status via alterations in metabolism and serotonin production.

## Discussion

Autism spectrum disorder (ASD) now affects approximately 1 in 161 children globally [16], and is continuing to rise in prevalence especially in developed countries. Moreover, children with ASD are commonly burdened with various gastrointestinal (GI) disorders [4] ranging from mild GI discomfort and constipation [49] to inflammatory bowel disease (IBD). Numerous studies have also reported significant alterations in the composition of gut microbes in ASD children burdened with GI disorders [12, 13, 28, 29], the dysbiosis of which is highly responsible for the development of GI disorders and IBD [7]. Approximately 40% of children with ASD also have elevated level of serotonin [2, 3], which might cause diarrhea and even altered mental status [11]. In this animal model study, we showed that modeling two environmental risk factors of autism —prenatal injection of poly I:C or valproic acid (VPA)—results in a distinct pattern of gut microbial dysbiosis that highly recapitulates those observed in children with ASD or patients with IBD (Fig. 2). We have also observed that ASD mice have gut microbial dysbiosis with decreased alpha-diversity compared to CTL mice, which is in line with few previous publications on gut microbial profiling of children with ASD [13] or patients with IBD [14]. This suggests that prenatal events could act as causal agents for microbial alteration responsible for IBD [50, 51], one of the most common comorbidities observed in ASD.

Our result on IBD-like microbial dysbiosis in two environmental risk factor models of ASD adds support to previous studies that have investigated how prenatal injection of poly I:C or VPA affects the GI tract in offspring later in life. Similar to patients with IBD [52], poly I:C offspring exhibit GI barrier defects such as increased permeability, and abnormal expression of tight junction proteins in the gut [9]. In addition, poly I:C offspring display significantly elevated level of plasma cytokines such as IL-2, IL-5, and IL-6 [53]. As for VPA, male VPA offspring have been shown to display epithelial loss in the ileum as well as signs of neutrophil infiltration in their gut [54], a phenomenon frequently observed in clinical cases of IBD [55, 56]. Prenatal VPA injection in rats resulted in reduced thickness of the mucosa and muscle layers of GI tract in the offspring, which was accompanied by significant changes in the morphologies of GI epithelial cells such as atrophy, weak cytoplasm staining, and distracted arrangement of chief cells; moreover, the GI transit index was significantly decreased in the VPA offspring, indicating functional deficit of GI in response to prenatal VPA injection [57]. Taken together, previous studies have shown that prenatal administration of environmental risk factors of ASD can induce IBD-like symptoms in the offspring, and thereby suggest possible connection between ASD and IBD at the tissue level.

Out of the 259 taxa tested, the most prominently and uniquely altered taxa was *Prevotella*, a fermenter of plant polysaccharides. For this reason, it has been suggested that decrease in *Prevotella* in the intestine might be associated with various digestive disorders [13]. Accordingly, clinical reports have shown that *Prevotella* is significantly decreased in both autistic individuals [13] and IBD patients [15], indicating that changes in the relative abundance of *Prevotella* might be a key factor that links ASD and IBD. *Prevotella* has also recently attracted attention due to its distinct pattern of abundance in response to diet, in that it is highly prevalent in rural Africa and significantly reduced in the Western world [58], the latter of which harbors countries with one of the most highest incidence of ASD [16]. Notably, our present results bring a new insight to the relationship between ASD and *Prevotella*, in that prenatal environmental risk factors of ASD may render the host’s enteric environment inhospitable for the growth of *Prevotella*, an effect which seems to have overcome the controlled dietary environment in our study setting. It would be of potential value to test if colonization of *Prevotella* or administration of prebiotics aimed at increasing its prevalence might alter the inflammatory status of ASD mice or even its behavioral abnormalities.

We also showed that microbial dysbiosis is likely to affect the host’s health status via alteration of its metabolic pathways (Fig. 4a). A variety of metabolic disturbances has been reported in ASD [41, 42, 59–65] and IBD [43, 51, 66–69], with a possible relationship to the etiology or progression of those conditions. One example is the elevated level of lipopolysaccharide (LPS) and bacterial endotoxins: in ASD, an elevation of circulating level of bacterial endotoxin in ASD patients compared to healthy controls (HCs) was reported [45], and it was repeatedly demonstrated in murine models that early life exposure to LPS results in ASD-like behavioral abnormalities via inflammatory responses [70, 71]. With regards to IBD, endotoxemia was also reported in a major subset of patients with ulcerative colitis or Crohn’s disease [46], as well as serum markers of LPS exposure in pediatric IBD patients [66]. Interestingly, ASD mice also showed an elevated level of metabolic pathways associated with LPS: “Lipopolysaccharide biosynthesis”, “Lipopolysaccharide biosynthesis proteins”, and “Bacterial toxins” (Fig. 4a, purple box). Accordingly, one research group hypothesized that toxin-producing harmful gut bacterial species such as *Desulfovibrio* might contribute to ASD [29], and later reported that *Desulfovibrio* was found more frequently in the stools of autistic children compared to HCs [28]. Similarly, we also observed that the relative abundance of *Desulfovibrio* is significantly increased in both poly I:C and VPA mice (Fig. 2d). Taken together, our results link poly I:C- and VPA-induced microbial dysbiosis with endotoxemia implicated in both ASD and IBD, thereby lending more support to the microbial basis of ASD etiology and pathogenesis.

Hyperserotonemia in autistic individuals was first reported more than fifty years ago [72], and has been continued to be described in major subsets of autistic children to the point that researchers have suggested elevated serum serotonin as a possible cause or aggravator of ASD-like behavioral phenotypes [1, 3]. One study also proposed that serotonin pathway can be utilized as a biomarker for autism diagnosis [2], although the underlying cause for such aberration remained unclear. Recently, expression of a variant in serotonin transport gene (SLC6A4) in mice was shown to result in hyperserotonemia and ASD-related behavioral abnormalities [73]. However, no single genetic variant or mutation related to ASD accounts for more than 1-2% of the total affected population [74], a figure falling far short of the prevalence of hyperserotonemia (40%). Instead, we here report for the first time that modeling environmental risk factors of ASD in mice resulted in a significantly elevated level of serum serotonin (Fig. 4c), and that it is accompanied by alterations in bacterial metabolic pathway related to the production of SCFAs, which are known inducers of serotonin production [47]. We also show that the rise in serum serotonin was accompanied by an increased abundance of *Sporanaerobacter* of Clostridia class, a genus that forms spores and produces SCFAs [48]. This genus carries relevance in that a recent publication, through the use of germ-free mice, has skillfully demonstrated the role of spore-forming bacteria on the production of serotonin [10]. Thus, our results suggest that the widespread prevalence of hyperserotonemia in children with ASD might have a microbial basis.

In our study, it is possible that the metabolism and gut microbial status of the pre-weaned pups was largely affected by their mothers, possibly through milk ingestion. However, whereas Prevotellaceae was significantly reduced in the feces of offspring (Fig. 3a), the same phenomenon was not observed in the feces of their mothers (Additional file 2: Figure S2). Also, whereas serum from offspring right before weaning showed significantly increased amount of serotonin (Fig. 4c), their mothers did not show any statistically significant increase in their serum serotonin at any time point (Additional file 3: Figure S3). These results argue against the possibility that the mothers were the primary target of poly I:C and VPA injection in terms of microbial dysbiosis and hyperserotonemia.

A few papers have argued that the gut microbial composition of children with ASD do not differ significantly from those of HCs or siblings [75, 76]. The apparent discordances among microbiota studies on ASD children might stem from differences in sampling and sequencing methods or cohort designation regarding autism severity and the presence of GI disorders. Nevertheless, mounting clinical evidence shows that children with ASD do suffer from various GI disorders and IBD [4, 61, 77], and since microbial dysbiosis accounts for a significant part of the etiology of those conditions [7], gut microbiota in ASD warrants further study with a focus on adjusting for the high degree of inter-individual variability.

Microbiota as a research area is complex and rapidly growing, and its apparent involvement in ASD calls for targeted investigation of its relationship with the pathogenesis of the disorder. Our present study using animal model shows that (1) prenatal exposure to environmental risk factors is able to alter the abundances of many gut microbial taxa later in life, and that (2) such dysbiosis carries the possibility of directly affecting the host’s health status via metabolic changes and serotonin pathway. Future research might benefit from focusing on the role of *Prevotella* in ASD and IBD, or describing in detail how ASD and IBD cause significant decreases in its abundance. We also suggest that poly I:C and VPA mouse are promising models for studying microbial dysbiosis in ASD, which might aid in developing a therapeutic regimen for ASD and/or IBD based on microbial manipulation.

## Additional files

Additional file 1: Figure S1. ASD-related behavioral abnormalities in ASD mice. (a) Representative movement trajectories of CTL and ASD mice in 10-min sessions. Black square denotes the center area measured for exploratory behavior. (b) Comparison of total distance moved (cm) in CTL and ASD mice. (c) Comparison of time spent in the center (black box) (s) in CTL and ASD mice. *p < 0.05 vs. CTL. (d) Representative movement trajectories of CTL and ASD mice in a 3-chamber apparatus in 10-min sessions. The upper row shows experimental session testing for preference for the social cue (S1; stranger 1) over non-social cue (Ob; object) preference, and lower row shows the session testing for preference for the novel social cue (S2; stranger 2) over familiar social cue (S1; stranger 1). (e and f) Bar graph representation of time spent at stranger 1 over an object (e) and time spent at stranger 2 over stranger 1 (f). *p < 0.05 vs. CTL. *n* = 7-10 in all experiments. (PDF 578 kb)

Additional file 2: Figure S2. Relative abundance of Prevotellaceae in mother mice. n.s. = not significant. *n* = 10-12. (PDF 81 kb)

Additional file 3: Figure S3. Serum serotonin level in mother mice. n.s. = not significant. *n* = 5 each. (PDF 192 kb)

Additional file 4: Table S1. List of microbial taxa significantly altered in poly I:C and VPA mice. Only the taxa with more than 20% changes in abundance relative to CTL are shown. (XLSX 15 kb)

Additional file 5: Table S2. List of metabolic pathways significantly altered in ASD mice. Only the pathways with more than 5% changes relative to CTL are shown. (XLSX 14 kb)
