# Supplementary figures and images for: Modeling environmental risk factors of autism in mice induces IBD-related gut microbial dysbiosis and hyperserotonemia
Source: Mol Brain. 2017 Apr 20;10:14. doi: 10.1186/s13041-017-0292-0 (PMC5399341; doi:10.1186/s13041-017-0292-0)

**A**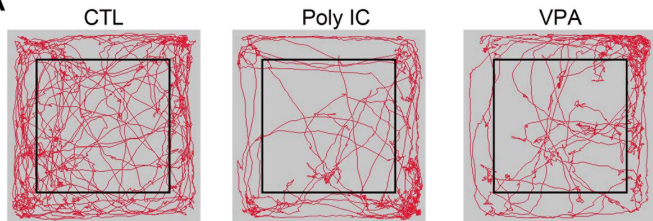**B**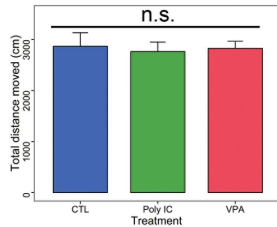**C**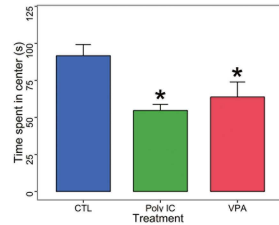**D**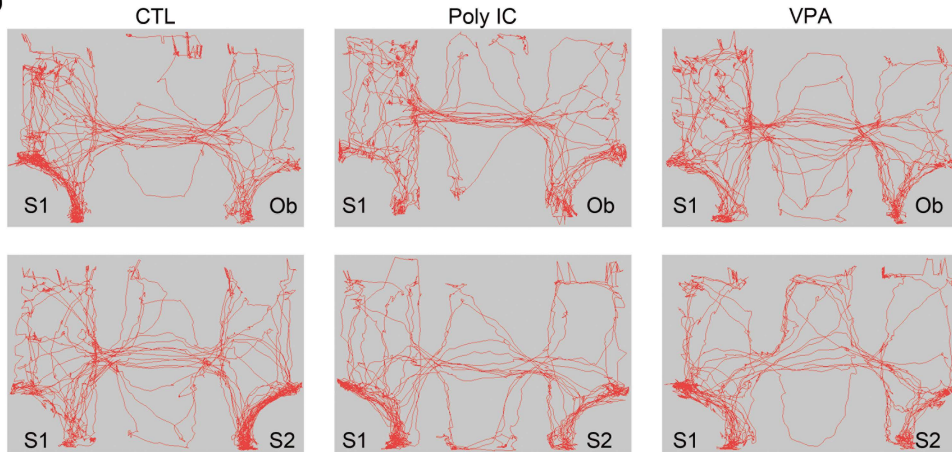**E**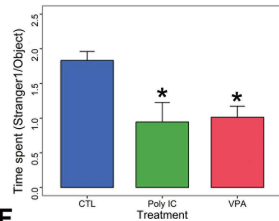**F**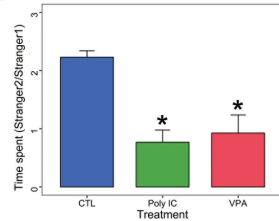

Supplement: Supplementary file 1 — ASD-related behavioral abnormalities in ASD mice. (a) Representative movement trajectories of CTL and ASD mice in 10-min sessions. Black square denotes the center area measured for exploratory behavior. (b) Comparison of total distance moved (cm) in CTL and ASD mice. (c) Comparison of time spent in the center (black box) (s) in CTL and ASD mice. *p < 0.05 vs. CTL. (d) Representative movement trajectories of CTL and ASD mice in a 3-chamber apparatus in 10-min sessions. The upper row shows experimental session testing for preference for the social cue (S1; stranger 1) over non-social cue (Ob; object) preference, and lower row shows the session testing for preference for the novel social cue (S2; stranger 2) over familiar social cue (S1; stranger 1). (e and f) Bar graph representation of time spent at stranger 1 over an object (e) and time spent at stranger 2 over stranger 1 (f). *p < 0.05 vs. CTL. n = 7-10 in all experiments. (PDF 578 kb) [file 13041_2017_292_MOESM1_ESM.pdf]

# Prevotellaceae

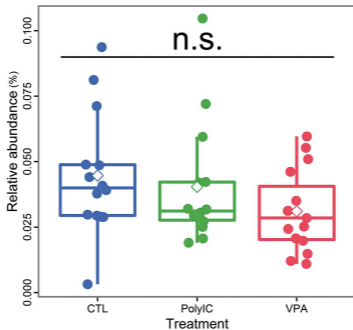

Supplement: Supplementary file 2 — Relative abundance of Prevotellaceae in mother mice. n.s. = not significant. n = 10-12. (PDF 81 kb) [file 13041_2017_292_MOESM2_ESM.pdf]

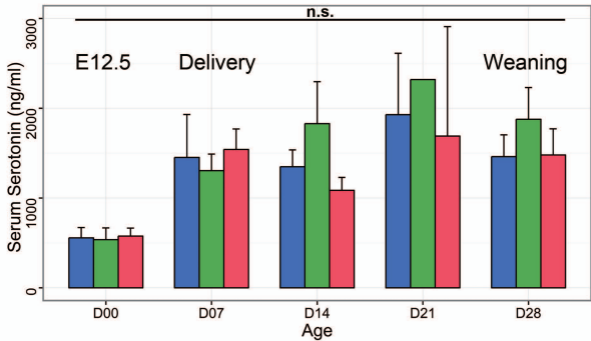

Supplement: Supplementary file 3 — Serum serotonin level in mother mice. n.s. = not significant. n = 5 each. (PDF 192 kb) [file 13041_2017_292_MOESM3_ESM.pdf]
